# Parent/carers' opinions about COVID‐19 vaccination for children with chronic lung diseases

**DOI:** 10.1002/hsr2.410

**Published:** 2021-10-01

**Authors:** Nusrat Homaira, Mei Chan, Louisa Owens, Amanda Thomsen, Melinda Gray, Sandra Chuang, Bernadette Prentice, Yvonne Belessis, Saiful Islam, Juliet Foster, Adam Jaffe

**Affiliations:** ^1^ Discipline of Paediatrics, School of Women's and Children's Health, Faculty of Medicine University of New South Wales Sydney New South Wales Australia; ^2^ Respiratory Department Sydney Children's Hospital Randwick New South Wales Australia; ^3^ School of Population Health University of New South Wales Sydney New South Wales Australia; ^4^ Clinical Management Group Woolcock Institute of Medical Research, University of Sydney Sydney New South Wales Australia

## 1 BACKGROUND

On April 9, 2021, the Pfizer Inc. and BioNTech SE requested the emergency authorization of Pfizer‐BioNTech COVID‐19 vaccine to be used in adolescents in the USA^1^ and the vaccine is now being used in teenagers in different parts of the world under emergency authorization.

When a COVID‐19 vaccine becomes available for wider use in other pediatric populations across the world, it is likely that vulnerable children such as those with an underlying chronic lung disease (CLD) may be prioritized, as they have been in adult populations. Children with CLDs including those with asthma, cystic fibrosis (CF), non‐CF bronchiectasis, and other chronic/congenital conditions are at higher risk of severe viral respiratory infection compared to children without CLDs and are often prioritized for other immunization against significant respiratory infections including influenza and pneumococcal disease.^2^, ^3^

Therefore, we conducted a survey among parents/carers of children with CLDs to determine their reasons for accepting or rejecting COVID‐19 vaccines for their children.

## 2 METHODS

### 2.1 Study design and population

During November 2020 to February 2021, we conducted an online survey at Sydney Children's Hospital (SCH), Randwick. The SCH is one of Australia's leading tertiary pediatric healthcare entities and cares for more than 600 children with CLDs including asthma, CF, congenital diaphragmatic hernia (CDH), tracheo‐esophageal fistula (TOF), non‐CF bronchiectasis, primary ciliary dyskinesia (PCD), and other respiratory conditions. We reviewed the electronic medical records to collate a list of all children with CLDs who received care from SCH during 2015 to 2020. Parents/carers of all children with CLDs identified from the medical records were eligible to participate in the study. Parents/carers of children without chronic lung disease or who did not speak English were not included in the survey.

An email with the URL link and QR code to the online survey was sent to all eligible participants who had valid email addresses. Mails with the study URL address and QR code were posted to residential addresses where email addresses were not available. Flyers of the study including the URL address and QR code were also displayed in the respiratory and outpatient departments of the hospital.

#### 2.1.1 The survey questionnaire

The survey included 14 closed‐ended questions based on three major domains: (a) demographic data, (b) factors associated with vaccine acceptance (comprised of 13 items), and (c) factors associated with vaccine refusal (comprised of 16 items). For each question around predictors for vaccine acceptance and refusal, responses were recorded on a five‐point Likert scale (strongly agree, agree, unsure, disagree, and strongly disagree). The question “If a vaccine against COVID‐19 becomes available in the future, will you get your child vaccinated?” was utilized to determine the likelihood of vaccination. Likelihood to vaccinate was defined as yes if a response was very likely or likely and no if a response was somewhat unlikely and unlikely. Respondents who were likely to vaccinate their children with CLDs then responded to questions associated with reasons for accepting COVID‐19 vaccine for their children (Table 1). Similarly, respondents who were unlikely to vaccinate responded to questions associated with reasons for refusing COVID‐19 vaccine for their children with CLDs (Table 2). Respondents who responded unsure to whether they would get their child vaccinated against COVID‐19 responded to both questions around reasons for accepting the vaccine and for refusing the vaccine.

**TABLE 1:** Reasons for wanting to get children with chronic lung diseases vaccinated against COVID‐19

	Agree	Unsure	Disagree	Total n
The vaccine can prevent my child from getting COVID‐19	144 (84%)	25 (15%)	2 (1%)	171
The vaccine will be safe for my child	106 (63%)	60 (30%)	1 (1%)	167
My child is at greater risk of COVID‐19 infection than a child without chronic lung condition	136 (81%)	24 (14%)	7 (5%)	167
My child has higher risk of COVID‐19 infection, vaccinating my child will protect people around my child from catching COVID‐19 infection	102 (61%)	40 (24%)	22 (13%)	166
I would follow my child's specialist/pediatrician advice to get my child vaccinated	157 (94%)	10 (6%)	0 (0%)	167
I would follow my child's GP advice to get my child vaccinated	134 (80%)	22 (13%)	11 (7%)	167
I would follow the recommendations of the State Health Department for all children with a lung condition to be vaccinated	138 (82%)	23 (14%)	8 (5%)	169
If the vaccine is free	119 (72%)	22 (13%)	21 (13%)	165
If the vaccine is offered for my child when my child visits his/her specialist/pediatrician for regular follow‐up	136 (81%)	27 (16%)	5 (3%)	168
If vaccinating my child against COVID‐19 allows me/my child to travel or visit public and private places within Australia – such as schools, other people's home	147 (87%)	14 (8%)	8 (5%)	169
If vaccinating my child against COVID‐19 allows me/my child to travel or visit public and private places overseas	138 (82)	23 (14%)	8 (5%)	169

**TABLE 2:** Reasons for not wanting children with chronic lung diseases vaccinated against COVID‐19

	Agreed	Unsure	Disagreed	Total n
COVID‐19 is not a severe disease	2 (5%)	13 (31%)	27 (64%)	42
The vaccine may not work and may give my child COVID‐19 disease	12 (31%)	14 (36%)	13 (33%)	39
The vaccine may give my child an illness other than COVID‐19	20 (50%)	9 (23%)	11 (28%)	40
Children with a lung condition are not at risk of COVID‐19 disease	2 (5%)	11 (28%)	27 (68%)	40
The shot may be painful for my child	7 (17%)	1 (3%)	32 (80%)	40
If it is not convenient for me, I will not take my child for vaccination	3 (7%)	2 (5%)	35 (78%)	40
There could be side effects from the COVID‐19 vaccine	34 (83%)	5 (12%)	2 (5%)	41
I do not believe in vaccines in general	3 (8%)	3 (8%)	33 (85%)	39
I do not believe in vaccines for viruses that are like the flu	6 (15%)	5 (13%)	29 (73%)	40
I prefer my child to fight off COVID‐19 naturally, without using a vaccine	2 (5%)	12 (30%)	26 (65%)	40
I prefer to keep my child at home to reduce the risk of getting COVID‐19	13 (32)	4 (10%)	23 (58%)	40
I do not believe it will be properly tested in children before being rolled out to the public	17 (43%)	10 (25%)	2 (5%)	40
I will rather get the vaccine myself and protect my child from getting the infection	5 (12%)	20 (50%)	15 (38%)	40
My child is too young to be vaccinated	5 (12%)	9 (23%)	26 (65%)	40

### 2.2 Statistical analysis

We used descriptive statistics such as frequency and proportions where appropriate. For purpose of analysis responses such as agree/strongly agree and disagree/strongly disagree were grouped as agree or disagree, respectively. Area‐level socioeconomic status (SES) was assessed using the Australian Bureau of Statistics Socio‐Economic Indexes for Areas (SEIFA) Index of Relative Socioeconomic Advantage and Disadvantage (IRSAD), based on the child's residential postcode. SES was according to the postcode's NSW percentiles.^4^ All analyses were performed in STATA version 15.0, StataCorp.

### 2.3 Ethics approval and consent

The study was approved by the ethics committee of the Sydney Children's Hospitals Network (2020/ETH02556). Information about the study including nature, purpose, and duration of the study was displayed on the front page of the online survey and included in the emails and the letters. Participants consented by checking the “YES” button in the survey.

## 3 RESULTS

We sent out 230 emails and 90 mails to 320 parents/carers of children with CLDs who had valid email and residential addresses listed in the medical records. Parents/carers of children with CLDs who came in for medical review to SCH during the study period were also able to participate in the survey by clicking the URL link and QR code to the online survey flyer available in the respiratory and outpatient departments of the hospital; however, as participation was completely anonymous, we were not able to ascertain the total number of parents who participated due to the email/mail invitations or the flyer invitation. In total, 207 parents/carers consented to participate but 202 parents/carers of 202 children with CLDs completed the survey and were included in the analysis. The background characteristics of the children with CLDs and their parents/carers who participated in the survey are presented in (Table S1). The vast majority of the parents/carers (152/198, 77%) said that they were likely to get their child with CLD vaccinated against COVID‐19, 9% (18/198) were not likely to get their child vaccinated and 14% (28/198) were unsure. In regard to the location of vaccination, either the general practitioner's (GP) clinic (105/181, 58%) or the hospital (65/181, 36%) was the preferred location to get their children with CLD vaccinated.

Parents/carers who were likely to have their child with CLD vaccinated stated that they would immunize the child against COVID‐19 if it was recommended by the child's pediatrician/specialist physician (94%,157/167) and/or general practitioner (GP) (80%, 134/167) (Table 1). On the other hand, parents/carers who were not willing to vaccinate their children with CLD against COVID‐19 stated that there could be side effects associated with the vaccine (83%, 34/41) and that the vaccine might not be tested properly in children before being rolled out to the public (68%, 27/40) (Table 2).

## 4 DISCUSSION

In our study, the vast majority (~80%) of the parents/carers of children with CLDs were willing to get their child vaccinated against COVID‐19. This is in stark contrast to previous studies that have reported that only around 40% parents/carers of children with chronic condition intended to get their children vaccinated against influenza^5^ and 69% of mothers intended to vaccinate their children against COVID‐19.^6^ Risk perception is a major driver in health behavior and the fact that more than 80% of parents/carers in our study believed that children with CLDs are at greater risk of COVID‐19 infection than children without CLD may act as a key factor in increasing vaccine coverage in high‐risk children^7^ when a COVID‐19 vaccine will be rolled to wider pediatric populations.

However, one‐third of the parents (30%) who were likely to get their child vaccinated were unsure if the vaccine would be safe for the child. Concerns around the safety and side effects of the vaccine were also some of the major reasons cited by parents/carers who were not likely to get their child with CLD vaccinated and have been reported previously as well.^6^ In addition, half of the parents/carers who were not likely to get their child vaccinated were concerned about their child getting an illness other than COVID‐19 from the vaccine and one‐third of them were willing to keep their children at home to protect than from COVID‐19 rather than getting them vaccinated. These lines of evidence suggest that while there is consensus among parents/carers of children with CLDs regarding the severity of COVID‐19, the major issue around uptake of COVID‐19 vaccine for children with CLDs is probably around safety issues.^7^ These concerns might be further enhanced by the recent reports of unusual blood clots associated with COVID‐19 vaccine.^8^

The majority of the parents/carers reported that advice from the child's specialist/pediatricians or GP will positively impact their decision to get their child vaccinated. Healthcare worker's recommendation has been consistently identified as a major predictor of vaccine uptake for other diseases as well.^9^ Children with CLDs require ongoing follow‐up visits with their pediatricians/specialists or GPs. GPs and pediatricians could use scheduled follow‐up visits of children with CLDs to promote COVID‐19 vaccine and explain to their parents/carers how the rare side effects outweigh the benefits of the vaccine.^10^

One of the major limitations of our study is that the survey participants were recruited from one hospital; however, SCH is one of the largest tertiary hospitals in Sydney with a large diverse catchment population. Also, we do not know the characteristics of the parents/carers who did not participate in the survey and can be very different from those who did. Nevertheless, our study has identified important factors that can be used in designing public health messages to improve COVID‐19 vaccine uptake in this specific high‐risk group of children.

## FUNDING

There was no funding source specific to this study. Nusrat Homaira is supported by a research fellowship of the National Health and Medical Research Council of the Australian Government (GNT1158646). The authors have indicated they have no financial relationships relevant to this article to disclose.

## AUTHOR CONTRIBUTIONS

Conceptualization: Nusrat Homaira, Mei Chan, Melinda Grey, Louisa Owens, Sandra Chuang, Amanda Thomsen, Bernadette Prentice, Yvonne Belessis, Juliet Foster, Saiful Islam and Adam Jaffe

Methodology: Nusrat Homaira, Mei Chan, Melinda Grey, Louisa Owens, Sandra Chuang, Amanda Thomsen, Bernadette Prentice, Yvonne Belessis, Juliet Foster and Adam Jaffe

Project administration: Nusrat Homaira, Mei Chan, Melinda Grey, Louisa Owens, Sandra Chuang, Amanda Thomsen, Bernadette Prentice

Data curation: Mei Chan

Formal Analysis: Mei Chan

Writing—Review and Editing: Nusrat Homaira, Mei Chan, Melinda Grey, Louisa Owens, Sandra Chuang, Amanda Thomsen, Bernadette Prentice, Yvonne Belessis, Juliet Foster, Saiful Islam and Adam Jaffe

Writing—Original Draft: Nusrat Homaira

All authors have read and approved the final version of the manuscript.

Nusrat Homaira had full access to all of the data in the study and takes complete responsibility for the integrity of the data and the accuracy of the data analysis.

## CONFLICT OF INTEREST

The authors have no conflicts of interest relevant to this article to disclose.

## Supporting information


**Table S1.** Background characteristics of study participants
