# Supplementary material for: Parent/carers' opinions about COVID‐19 vaccination for children with chronic lung diseases
Source: Health Sci Rep. 2021 Oct 1;4(4):e410. doi: 10.1002/hsr2.410 (PMC8485617; doi:10.1002/hsr2.410)
Supplement: Supplementary file 1 — Table S1. Background characteristics of study participants [file HSR2-4-e410-s001.docx]

**Supplementary table 1: Background characteristics of study participants**

|  | **Total**  **N=202** | **n (%)** | **Likely to vaccinate**  **n/subtotal (%)**  **n=155** | **Unsure**  **n/subtotal (%)**  **n=22** | **Unlikely to vaccinate**  **n/subtotal (%)**  **n=19** |
| --- | --- | --- | --- | --- | --- |
| **Characteristics of the child with chronic lung disease** | | | | | |
| Gender (female) | 190 | 87(46) | 71/146 (37) | 7/25 (28) | 9/18 (50) |
| Median age in years (±SD) | 202 | 8.4 (5.1)^a^ | 8.6 (5.2) ^a^ | 7.4 (4.2) ^a^ | 9.1 (5.2)^a^ |
| Type of chronic lung disease | 199 |  |  |  |  |
| *Asthma* |  | 67 (34) | 51/155 (33) | 11/25 (44) | 5/19 (26) |
| *Cystic fibrosis* |  | 94 (47) | 74/155 (48) | 10/25 (40) | 10/19 (53) |
| *Congenital diaphragmatic hernia* |  | 21 (11) | 18/155 (12) | 2/25 (8) | 1/19 (5) |
| *Bronchiectasis* |  | 13 (7) | 9/155 (6) | 2/25 (8) | 2/19 (11) |
| *Other* |  | 4 (2) | 3/155 (2) | 0/25 (0) | 1/19 (5) |
| **Characteristics of the parents/carers of the child with chronic lung disease** | | | | |  |
| Gender (female) | 185 | 150 (81) | 114/143 (80) | 21/24 (88) | 15/18 (83) |
| Relationship to the child | 201 |  |  |  |  |
| *Mother* |  | 177 (88) | 135/155 (87) | 26/18 (93) | 16/18 (89) |
| *Father* |  | 23 (11) | 20/155 (13) | 1/28 (4) | 2/18 (11) |
| *Grandparent* |  | 1 (1) | 0/155 (0) | 1/28 (4) | 0/18 (0) |
| Age group | 190 |  |  |  |  |
| *≤ 29 years* |  | 15 (8) | 12/147 (8) | 2/25 (8) | 1/18 (6) |
| *30-39 years* |  | 55 (29) | 42/147 (29) | 8/25 (32) | 5/18 (28) |
| *40-49 years* |  | 98 (52) | 75/147 (51) | 14/25 (56) | 9/18 (50) |
| *≥50 years* |  | 22 (12) | 18/147 (12) | 1/25 (4) | 3/18 (17) |
| Education level | 187 |  |  |  |  |
| *Postgraduate education level ^b^* |  | 64 (34) | 51/145 (35) | 5/25 (20) | 8/17 (47) |
| *Undergraduate education level ^c^* |  | 106 (57) | 79/145 (54) | 18/25 (72) | 9/17 (53) |
| *Primary or Secondary education level* |  | 17 (9) | 15/145 (10) | 2/25 (8) | 0/17 (0) |
| Speak a language other than English at home (Yes) | 190 | 41 (22) | 27/147 (18) | 8/25 (32) | 6/18 (22) |
| SEIFA (Decile ranking) ^e^ | 189 | 7.9 (2.3)^a^ | 8.0 (2.4) ^a^ | 7.4 (2.0) ^a^ | 7.8 (2.4) ^a^ |
| **Do you agree children with lung conditions should be prioritized for vaccination against COVID-19?** | | | | | |
| Agree | 199 | 167 (84) | 140/153 (91.5) | 20/28 (71) | 7/18 (39) |
| Unsure |  | 28 (14) | 13/153 (8) | 8/28 (29) | 7/18 (39) |
| Disagree |  | 4 (2) | 0/153 (0) | 0/28 (0) | 4/18 (22) |
| **What is your preferred location to get the child vaccinated against COVID-19** | | | | | |
| *Hospital* | 181 | 66 (36) | 54/151 (36) | 11/28 (39) | 1/2 (50) |
| *GP clinic* |  | 105 (58) | 89/151 (59) | 15/28 (54) | 1/2 (50) |
| *Others ^d^* |  | 10 (1) | 8/151 (5) | 2/28 (7) | 0/2 (0) |

n=number of respondents; SEIFA= Socio-Economic Indexes for Areas;  ^a^ value in mean (SD); ^b^ Postgraduate qualifications including PhD, Master Degree, Graduate Diploma or Graduate Certificate; ^c^ Undergraduate qualification including Bachelor Degree Advanced Diploma, Diploma, Certificate III & IV or Certificate I & II; ^d^ Other locations including local health clinic, school, pharmacy or drive through clinic
